# Geometrically aware transformer for point cloud analysis

**DOI:** 10.1038/s41598-025-00789-7

**Published:** 2025-05-13

**Authors:** Siyuan Chen, Zhiwei Fang, Siyao Wan, Ting Zhou, Chunlin Chen, Meng Wang, Qianming Li

**Affiliations:** 1 School of Information Science and Engineering, Hunan Institute of Science and Technology, Yueyang, 414006 China; 2 Hunan Institute of Science and Technology, College of Mechanical Engineering, Yueyang, 414006 China; 3 Department of Engineering Surveying, Yueyang Institute of Water Resources and Hydropower Survey Planning and Design Co., LTD., Yueyang, 414004 China

**Keywords:** Computational science, Computer science, Information technology

## Abstract

With the increasing use of 3D point cloud data in autonomous driving, robotic perception, and remote sensing, efficient and accurate point cloud analysis remains a critical challenge. This study presents PointGA, a lightweight Transformer-based model that enhances geometric perception for improved feature extraction and representation. First, PointGA expands the original 3D coordinates into various geometric information, introducing more prior knowledge into the network. Second, a trigonometric position encoding suitable for point clouds is designed, which effectively enhances the expressive capability of positional information and performs preliminary feature extraction through pooling layers, significantly improving the model’s robustness across various tasks. Finally, a positional differential self-attention (PDA) mechanism with linear complexity is developed to optimize feature representation and achieve efficient computation. Experimental results demonstrate that PointGA achieves 87.6% overall accuracy on the ScanObjectNN dataset for classification and 66.2% mean intersection over union(mIoU) on the S3DIS Area 5 dataset for segmentation, outperforming existing methods. These findings highlight the model’s capability to balance efficiency and accuracy, offering a promising solution for point cloud analysis tasks.

## Introduction

Point cloud analysis has become a critical task in the field of 3D computer vision, with wide-ranging applications in autonomous driving^1–3^, robotics^4–6^, augmented reality^7–10^, and geospatial surveying^11–13^. Recent studies have further demonstrated the versatility of 3D point clouds in diverse domains such as plant phenotyping, structural health monitoring, and precision measurement. For instance, Zhou et al.^14^ utilized 3D point cloud data for automated phenotyping of Chinese Cymbidium seedlings, highlighting its potential in agricultural intelligence. In the field of civil engineering, Zhang et al.^15^ integrated point clouds and images into a digital twin framework to estimate the load-carrying capacity of cracked reinforced concrete beams. Similarly, Dong et al.^16^ applied a local-feature-based 3D point cloud stitching method for aero-engine blade measurement, emphasizing the importance of accurate alignment in industrial inspection tasks. These works collectively underscore the growing importance of point cloud processing across diverse fields. They further emphasize the need for robust point cloud analysis algorithms capable of supporting high-level scene understanding. Unlike traditional 2D images, point clouds not only accurately represent the 3D world but also capture the geometric features and spatial relationships of objects, which are essential for scene understanding. However, point cloud data is unordered, sparse, and often accompanied by noise, posing significant challenges for processing. Additionally, conventional Convolutional Neural Networks (CNNs) struggle with unstructured point cloud data due to their reliance on structured grid inputs, making it difficult to extract meaningful features. Addressing variations in point density, mitigating noise effects, and efficiently handling large-scale point clouds remain key challenges in this domain.

To tackle these challenges, researchers have proposed various deep learning-based approaches, which can be categorized into three main types: voxel-based, multi-view, and point-based methods. Voxel-based approaches, such as VoxelNet^17^, convert point clouds into regular voxel grids and apply 3D convolutions for feature extraction. However, voxelization often leads to the loss of fine-grained geometric details and suffers from increased computational costs^18^. Multi-view methods, such as MVCNN^19^, leverage 2D projections of point clouds, enabling the use of well-established 2D CNNs, but they struggle to capture complex spatial structures. While both voxel-based and multi-view methods offer advantages, they inherently involve information loss due to data transformation processes. As a result, researchers have turned to point-based methods, which directly process raw point cloud data and better preserve spatial relationships.

PointNet^20^ is a pioneering work in this field, as it directly processes point cloud data without the need for converting it into other formats such as voxel grids or images. PointNet utilizes symmetric functions to handle unordered point clouds, enabling both point-level feature learning and global feature extraction. Subsequently, PointNet++^21^ further improved performance by enhancing the ability to learn local features. DGCNN^22^ expanded the application scope of models by capturing local geometric relationships through dynamic graph structures. Recent studies, such as KPConv (14.3M)^23^ and PointMLP (12.6M)^24^, have introduced a significant number of parameters and complex computational operations to further enhance the accuracy and robustness of point cloud analysis. These methods predominantly utilize Multi-Layer Perceptrons (MLPs) to construct efficient feature extraction modules. MLPs have a simple and flexible structure and perform exceptionally well on small-scale data. However, as the data scale increases, MLPs exhibit significant limitations in capturing complex geometric structures and global features. This is especially pronounced when handling large-scale point clouds, where issues of computational efficiency and memory consumption become increasingly prominent.

Early point cloud processing methods often relied solely on 3D coordinate information. However, with the advancement of research, an increasing number of studies have begun to introduce richer geometric features, such as normal vectors, curvature, and neighborhood relationships^25–28^. GBN^29^ and EIP^30^ extend the 3D coordinate information by incorporating these richer geometric features, providing prior knowledge to the network and enhancing its ability to abstract complex features. Nonetheless, they also have certain limitations. First, GBN incurs high computational costs when processing large-scale point clouds, especially with the introduction of high-dimensional geometric information, which can lead to increased network complexity and affect inference speed. Second, although EIP enriches point cloud data through implicit representation, its effectiveness is limited when dealing with point cloud sparsity or non-uniform distributions, potentially making it challenging to capture fine-grained local geometric features adequately. While these methods enhance feature representation, they also introduce additional computational overhead and efficiency issues.

Recently, Transformer architectures^31^ have been explored for point cloud processing, leveraging position encoding and self-attention mechanisms to model long-range dependencies^32–38^. Since point clouds inherently lack explicit sequential information and there is no fixed order between points, the introduction of position encoding allows the model to perceive spatial relationships within the point cloud. Moreover, the self-attention mechanism can flexibly handle the relationships between points without relying on a fixed adjacency structure, thereby better capturing global contextual information. Classic models like PCT^39^ and Point Transformer^40^ have integrated the Transformer architecture to more effectively fuse local and global information, enhancing the performance of point cloud analysis tasks. Point-BERT^41^ draws inspiration from successful practices in natural language processing, proposing a Transformer-based self-supervised pretraining approach for feature learning in point clouds. By pretraining on large-scale unlabeled data, Point-BERT improves the performance of point cloud classification, segmentation, and other tasks. Additionally, Lai et al. proposed the Stratified Transformer^42^, which applies different sampling strategies to the neighboring points of each query point, enabling the model to achieve a larger receptive field at a lower computational cost, thus effectively balancing efficiency and accuracy when processing large-scale point clouds. SAT3D^43^, by introducing a slot attention mechanism, effectively aggregates and allocates features within the point cloud, improving the precision and robustness of segmentation tasks. However, despite the significant performance improvements offered by Transformers, the self-attention mechanism requires the computation of similarity between each point and all other points, resulting in a quadratic increase in computational complexity($O(N^2)$). This issue becomes particularly pronounced when handling large-scale point clouds, severely limiting the real-time capabilities and scalability of Transformers.

To address these limitations, this research proposes a lightweight Transformer network, termed PointGA, which integrates geometric-awareness to enhance efficiency. First, inspired by prior studies^27,29,30^, a parameter-free geometric information extension module has been introduced to expand raw 3D coordinates into a richer set of geometric features with minimal computational overhead. Second, a novel trigonometric position encoding module is designed to map point coordinates into a high-dimensional space, leveraging the periodic and continuous properties of trigonometric functions to capture spatial structures more effectively. This technique aligns with recent methods such as PointNN^44^ and SegNN^45^, where spatial embeddings improve feature representations. Finally, a positional difference self-attention (PDA) mechanism with linear complexity (*O*(*N*)) is developed to efficiently refine features while balancing computational cost and expressive capability. In summary, this research presents a simple yet effective framework, PointGA, which combines geometric information extension, trigonometric position encoding, and a low-cost self-attention mechanism to enhance point cloud analysis. The primary contributions of this research are summarized as follows:

- A novel method has been designed to extend spatial coordinate information into higher-dimensional geometric information, effectively enhancing the feature representation capability of point cloud data.
- A trigonometric position encoding module has been developed, which encodes the spatial coordinate information of the point cloud into a higher-dimensional space, thereby improving the model’s ability to perceive the relative positions between points.
- A low-cost self-attention mechanism with linear complexity has been designed to balance feature representation and computational efficiency.
- Extensive experiments have been conducted on multiple benchmark datasets, and the results demonstrate that the proposed network achieves significant performance with lower computational costs, validating the effectiveness and superiority of the proposed method.

The remainder of this paper is organized as follows. “Methods” provides a comprehensive introduction to the relevant technologies and the proposed PointGA architecture. “Experiments” presents the evaluation results of the method on multiple datasets. “Ablation study” analyzes the contributions of individual components through detailed experiments. “Discussion” evaluates PointGA’s efficiency, dataset-dependent performance variations, and potential improvements for real-world adaptability. Finally, “Conclusions” summarizes the findings and discusses potential future research directions.

## Methods

To illustrate how the Transformer mechanism in this study diverges from previous approaches, a brief review of classic Transformer types is conducted through mathematical formulations. An overview of PointGA is then provided, followed by a detailed description of the proposed geometric information extension module, the triangular function positional encoding module for initial feature extraction in conjunction with other information, and the self-attention module with linear complexity.

**Fig. 1:**
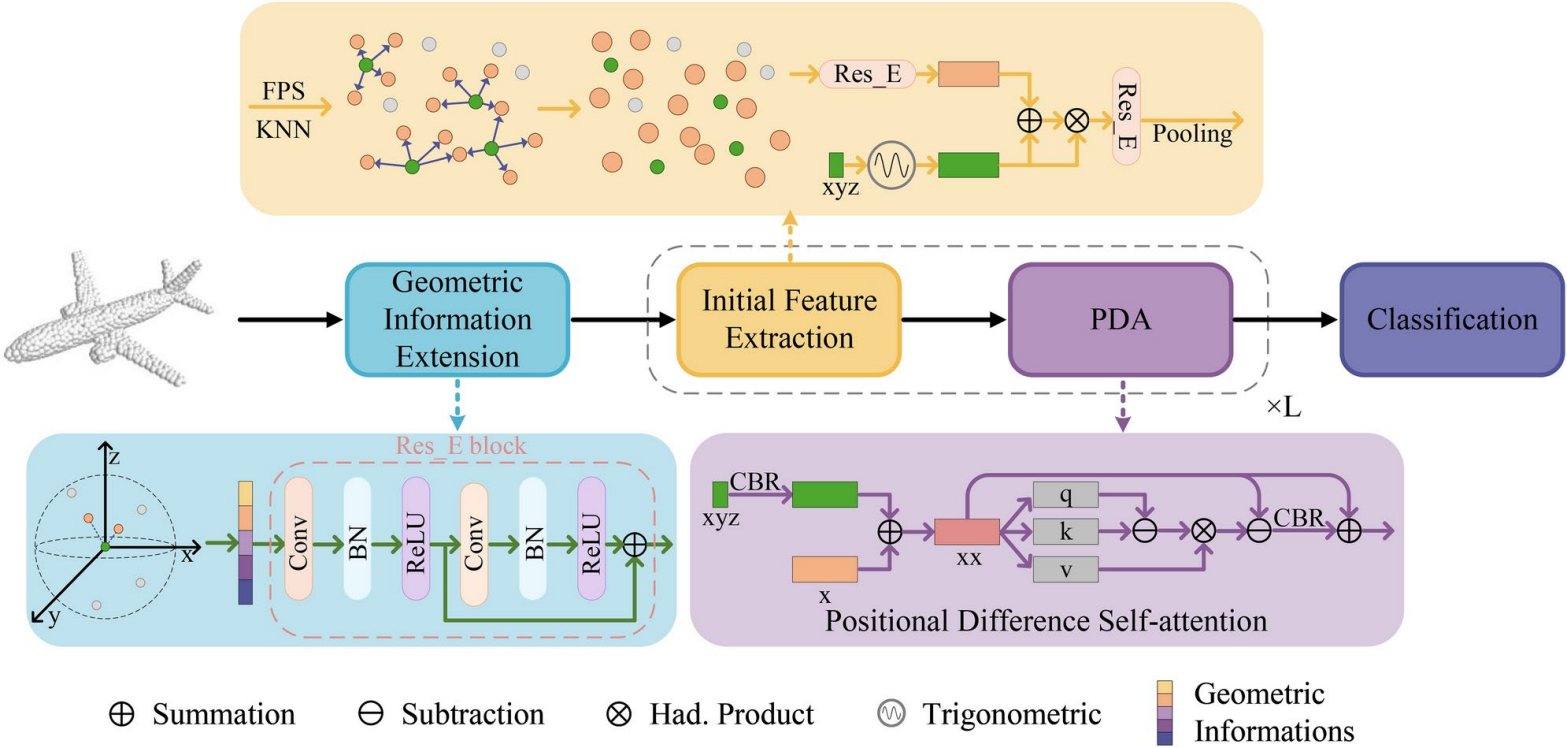
The framework diagram for PointGA in point cloud classification. The geometric information expansion module enriches the point cloud features, followed by position encoding through trigonometric functions applied to the point set processed by FPS and KNN. A pooling layer conducts preliminary feature extraction. Finally, the self-attention mechanism models spatial characteristics, using positional differences to emphasize relative positions among points and enhance local structure perception.

### Revisiting transformer for point cloud analysis

Transformers based on self-attention have been widely applied in point cloud analysis, achieving significant results. In general, attention operators can be divided into two categories: scalar attention ^31^ and vector attention ^46^.

Let $\mathscr {P}=\{{{p}_{i}}\mid i=1,\ldots ,N\}$ be a point set, where each point ${{p}_{i}}$ has three-dimensional coordinates $({{x}_{i}},{{y}_{i}},{{z}_{i}})$. Assume that the feature ${{f}_{i}}\in {{\mathbb {R}}^{D}}$ can be obtained through a mapping function $F({{p}_{i}})$:

1 $$\begin{aligned} {f_i} = F({p_i}), \end{aligned}$$

Then all the features can be represented as ${{\mathscr {F}}_{in}}=\{{{f}_{i}}\}_{i=1}^{N}$. The self-attention mechanism in the Transformer framework first maps input features into the query, key, and value representations:

2 $$\begin{aligned} Q = W_q \mathscr {F}_{in}, \quad K = W_k \mathscr {F}_{in}, \quad V = W_v \mathscr {F}_{in}, \end{aligned}$$

where $W_q, W_k, W_v \in \mathbb {R}^{D \times D}$ are learnable weight matrices.

Scalar attention can be expressed in the following general form:

3 $$\begin{aligned} {{\textbf{Y}}_{si}}=\sum \limits _{j=1}^{N} \mathscr {S}\big (Q_i^\top K_j + \delta \big ) V_j, \end{aligned}$$

where ${\textbf{Y}}_{si}$ denotes the output feature, $\delta$ represents a positional encoding function, and $\mathscr {S}$ is a normalization function such as *softmax*. Since scalar attention assigns a uniform attention weight to all feature dimensions, it lacks fine-grained control over individual dimensions, limiting its expressiveness.

In contrast, vector attention enables independent weighting of each feature dimension, capturing more complex relationships:

4 $$\begin{aligned} {{\textbf{Y}}_{vi}}=\sum \limits _{j=1}^{N} \mathscr {S} \big (\beta (\alpha (Q_i, K_j) + \delta )\big ) \odot V_j, \end{aligned}$$

where $\alpha$ is a relational function (e.g., multiplication), $\beta$ is a mapping function generating the attention vector, and $\odot$ denotes the Hadamard product.

Regardless of whether scalar or vector attention is used, the core computation involves obtaining *Q*, *K*, *V*, computing attention weights, and then performing a weighted sum over the values. The computational complexity of this process remains at least $O(N^2 \cdot D)$.

### Framework

**Fig. 2:**
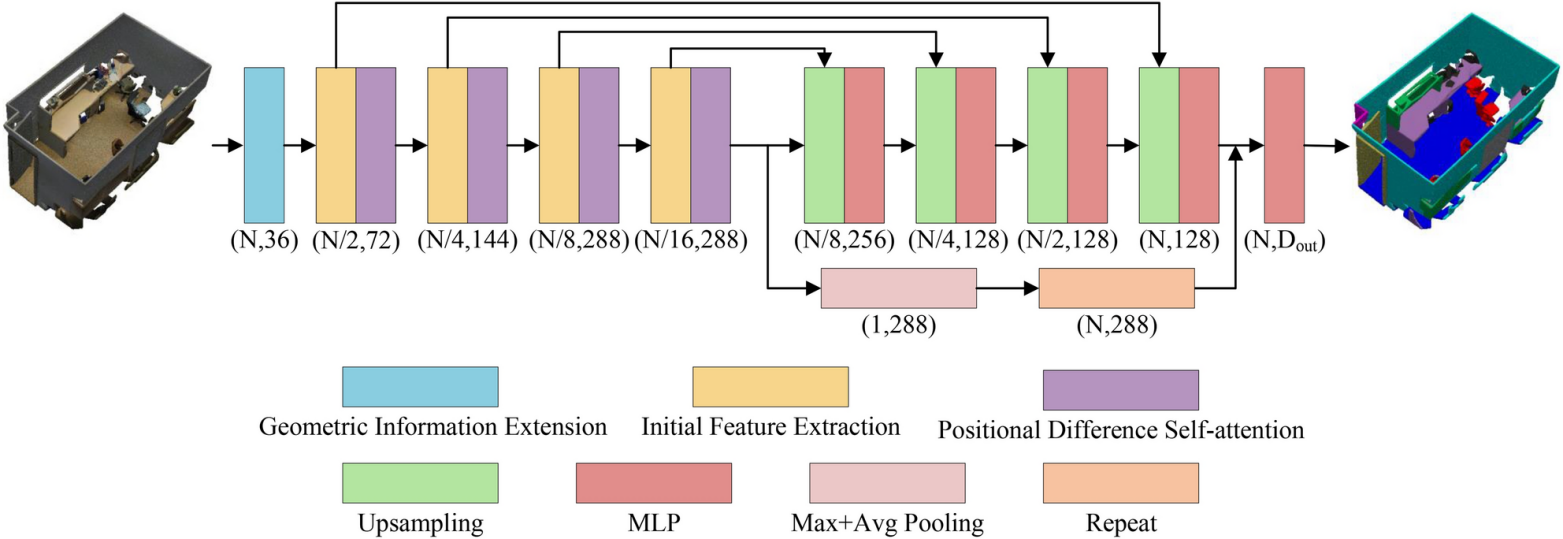
The framework diagram of PointGA in point cloud semantic segmentation. After multiple feature extractions, the features are integrated into a comprehensive representation for all points through two branches: one branch progressively propagates features to all points via upsampling and MLP, while the other branch undergoes pooling and is broadcasted *N* times. Finally, these two types of features are concatenated together for the subsequent semantic segmentation task.

During the Point cloud processing, to fully capture and understand the geometric properties of points and their interrelationships is crucial. Therefore, this research incorporates specific designs in the initial embedding and feature extraction phases, aiming to deeply extract geometric features and global contextual information from point cloud data to improve 3D point cloud classification and segmentation tasks. The overall architecture of the proposed PointGA method for classification and segmentation is illustrated in Figs. 1 and 2, respectively. To enable the network to acquire more effective prior information, geometric information extension is performed on the initial three-dimensional point cloud $\mathscr {P}=\{{{p}_{i}}\mid i=1,\ldots ,N\}$ (where each point ${{p}_{i}}$ consists of the Cartesian coordinates in xyz space, ${{p}_{i}}\in {{\mathbb {R}}^{3}}$), transforming it from 3D to a higher-dimensional space. MLP is employed as a means to enhance the model’s learning capability, followed by Batch Normalization (BN) layers and activation functions. Additionally, a residual structure^47^ is utilized to preserve more geometric information and mitigate the gradient vanishing problem.

In the feature extraction phase, the downsampling rate is set to 1/2 based on empirical settings, and a total of *L* feature extraction operations are conducted, with *L* fixed at 4 for the experiments.Unlike traditional PCT^39^, which solely stacks self-attention layers, PointGA first employs several simple non-learnable operators for initial feature extraction before utilizing the self-attention mechanism. Specifically, the sampled three-dimensional xyz coordinates are encoded using trigonometric functions, mapping them into a periodic high-dimensional feature space. Subsequently, the mapped high-dimensional positional information is combined with features enhanced through residual connections, and a weighted operation is applied to increase the network’s sensitivity to subtle variations in local geometric structures, along with pooling for preliminary feature extraction. Finally, the designed low-cost positional differential self-attention further enhances local feature extraction, improving the network’s ability to model global relationships.

### Geometric information extension

**Fig. 3:**
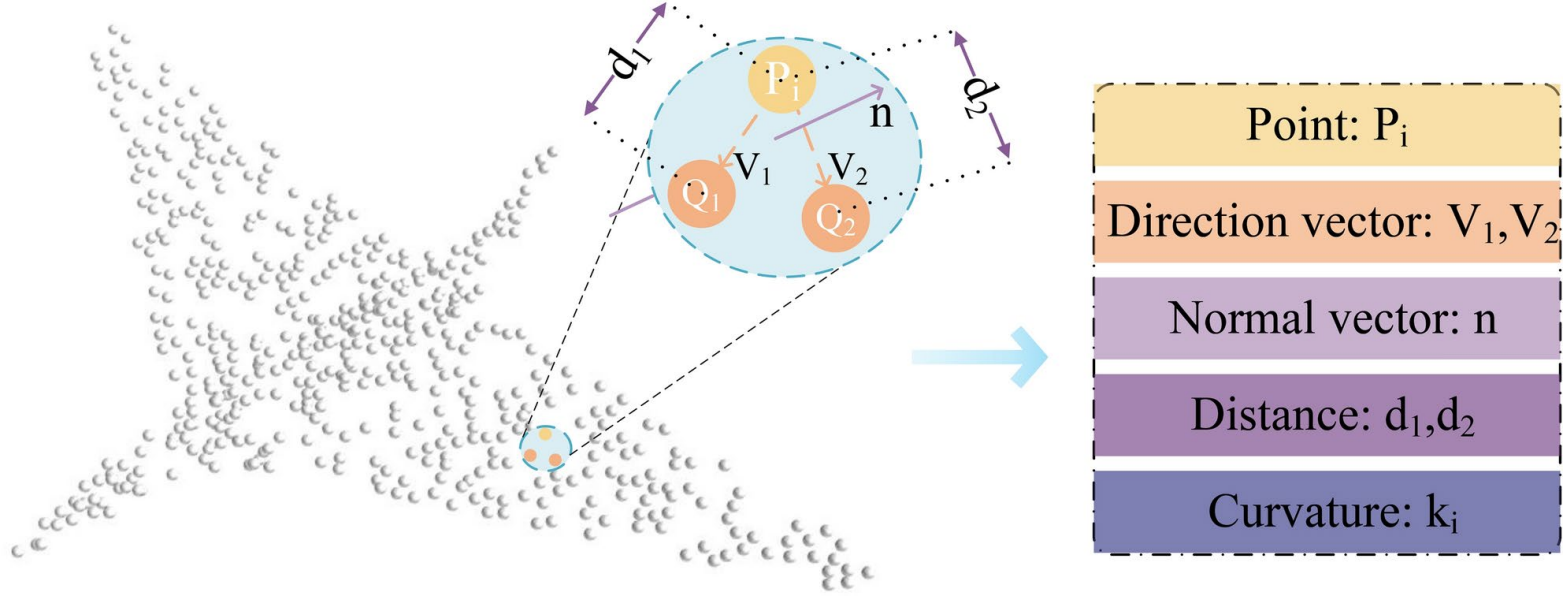
Geometric information extension module. The original 3D point $P_i$ is expanded to include four new types of geometric information: direction vectors, normal vectors, distances, and curvature.

To enhance the model’s understanding of complex spatial structures, as shown in Fig. 3, the single spatial attribute of the original point cloud is expanded into rich geometric descriptors. For each point $P_i$ in the point cloud, two neighboring points $Q_1$ and $Q_2$ are selected, and the direction vectors from $P_i$ to $Q_1$ and $Q_2$ are calculated:

5 $$\begin{aligned} \vec {V}_1 = Q_1 - P_i, \end{aligned}$$

6 $$\begin{aligned} \vec {V}_2 = Q_2 - P_i. \end{aligned}$$

The direction vectors help the network capture the relative positional relationships between points. Utilizing $\vec {V}_1$ and $\vec {V}_2$, the normal vector of this local plane $\vec {n}_i$ can be determined:

7 $$\begin{aligned} \vec {n}_i = \vec {V}_1 \times \vec {V}_2. \end{aligned}$$

This assists in improving the network’s perception of local geometric shapes. After calculating the normal vectors for all points, let us assume the neighborhood of each point $P_i$ is $\mathscr {N}(P_i)$. For the point $P_i$ and each point $P_j$ in its neighborhood, the change in normal vectors is computed:

8 $$\begin{aligned} \Delta \vec {n}_{ij} = \vec {n}_i - \vec {n}_j, \end{aligned}$$

where $\vec {n}_i$ and $\vec {n}_j$ are the normal vectors of points $P_i$ and $P_j$, respectively. The curvature $\kappa _i$ is estimated using the changes in the angles between the normal vectors and the distances between points:

9 $$\begin{aligned} \kappa _i = \frac{1}{|\mathscr {N}(P_i)|}\sum _{P_j \in \mathscr {N}(P_i)}{\frac{\Vert \Delta \vec {n}_{ij}\Vert }{\Vert P_i - P_j\Vert }}, \end{aligned}$$

where $\Vert \cdot \Vert$ denotes the norm, $|\mathscr {N}(P_i)|$ is the number of points around $P_i$, and $\kappa _i$ reflects the rate of change of the geometric shape, which aids the network in recognizing complex geometric structures.

Additionally, the distances $d_1$ and $d_2$ from point $P_i$ to the two neighboring points are calculated:

10 $$\begin{aligned} d_1 = \Vert Q_1 - P_i\Vert , \end{aligned}$$

11 $$\begin{aligned} d_2 = \Vert Q_2 - P_i\Vert . \end{aligned}$$

These distances help the network perceive local scale information of the point cloud. A residual combination of MLP, batch normalization, and activation functions is employed to further enhance the network’s ability to extract high-level features:

12 $$\begin{aligned} \mathscr {Y}(\cdot ) = \tau (BN(c_{1 \times 1}(\cdot ))) + \tau (BN(c_{1 \times 1}(\tau (BN(c_{1 \times 1}(\cdot )))), \end{aligned}$$

where $c_{1 \times 1}(\cdot )$ denotes the MLP, $\tau$ is the activation function, and $BN$ is the batch normalization layer.

### Initial feature extraction

For each stage of the feature extractor, the input point cloud representation from the previous stage is denoted as $\{{{p}_{i}},{{f}_{i}}\}_{i=1}^{N}$, where ${{p}_{i}}\in {{\mathbb {R}}^{1\times 3}}$ and ${{f}_{i}}\in {{\mathbb {R}}^{1\times C}}$ represent the spatial coordinates and features of point $i$, respectively. A subset of local center points is first selected using Furthest Point Sampling (FPS), with the downsampling rate set to $1/2$:

13 $$\begin{aligned} \{{{p}_{c}},{{f}_{c}}\}_{c=1}^{\frac{N}{2}}=\text {FPS}\left( \{{{p}_{i}},{{f}_{i}}\}_{i=1}^{N} \right) . \end{aligned}$$

Next, KNN is employed to select $k$ nearest neighbors for each center point to construct local regions:

14 $$\begin{aligned} \{{{p}_{{{i}_{k}}}}\}_{k=1}^{k}=\text {KNN}({{p}_{c}},\{{{p}_{i}}\}_{i=1}^{N}), \end{aligned}$$

To facilitate understanding, the set ${\mathscr {N}}_{c}=\{{{p}_{{{i}_{k}}}}\}_{k=1}^{k}$ is defined. To enable each point to obtain a larger receptive field, feature expansion is first performed by appending the center point’s features ${{f}_{c}}$ along the feature dimension to its neighboring points ${{f}_{k}}$:

15 $$\begin{aligned} {{f}_{ck}}=\text {Concat}({{f}_{c}},{{f}_{k}}),\text { for }k\in {{\mathscr {N}}_{c}}. \end{aligned}$$

As discussed in the context of Transformers, trigonometric function embeddings help the model learn relative positional information. The key idea is to encode the spatial position of each point in a higher-dimensional space using trigonometric functions like sine and cosine. Specifically, different scales of frequencies are introduced for the trigonometric functions, allowing each point to generate rich positional information. The point $p_{i}=({x_{i}, y_{i}, z_{i}}) \in \mathbb {R}^{1 \times 3}$ is embedded into $D$ dimensions, effectively representing each axis as $f_{i}^{x}, f_{i}^{y}, f_{i}^{z} \in \mathbb {R}^{1 \times \frac{D}{3}}$:

16 $$\begin{aligned} \text {TriE}({{p}_{i}})=\text {Concat}(f_{i}^{x},f_{i}^{y},f_{i}^{z})\in {{\mathbb {R}}^{1\times D}}. \end{aligned}$$

To enhance the model’s ability to capture geometric relationships across scales, each axis (x, y, z) is transformed into a set of sine and cosine functions based on the point’s coordinates. For example, taking the x-axis, trigonometric encoding is applied to the x-coordinate for each channel $c \in \{0, 1, 2, \ldots , \frac{D}{6}\}$. To better capture features at varying scales within complex geometries, the original encoding interval is mapped, expanding the channel range from $[0, \frac{D}{6}]$ to $[- \frac{D}{12}, \frac{D}{12}]$, enabling the model to more effectively represent positional information across different scales:

17 $$\begin{aligned} f_{i}^{x}[2c]=\sin \left( {{x}_{i}}/{{\alpha }^{\frac{12(2c-D/12)}{D}}} \right) , \end{aligned}$$

18 $$\begin{aligned} f_{i}^{x}[2c+1]=\cos \left( {{x}_{i}}/{{\alpha }^{\frac{12(2c-D/12)}{D}}} \right) , \end{aligned}$$

where $\alpha$ is the frequency scaling factor, set to $\alpha = 1000$ in the experiments. This adjustment enables the utilization of multiple frequencies to encode the input point cloud, thereby more effectively representing the geometric relationships between points. Subsequently, the encoded positional coordinates are applied to weight their corresponding features:

19 $$\begin{aligned} f_{ck}^{w}=\left( \begin{matrix} {{f}_{ck}}+\text {TriE}({{p}_{k}}) \\ \end{matrix} \right) \odot \text {TriE}({{p}_{k}}), \end{aligned}$$

where $f_{ck}^{w}$ represents the weighted features, and $\odot$ denotes the Hadamard product. Following the enhancement of $f_{ck}^{w}$ through a residual block, both max pooling and average pooling are utilized for feature aggregation. The max pooling captures the most salient local features, while the average pooling retains global information, thus providing a balance between local details and global context:

20 $$\begin{aligned} f_{c}^{a}=\text {MaxP}\left( {{\{f_{cj}^{w}\}}_{j\in {{\mathscr {N}}_{c}}}} \right) +\text {AveP}\left( {{\{f_{cj}^{w}\}}_{j\in {{\mathscr {N}}_{c}}}} \right) , \end{aligned}$$

where $f_{c}^{a} \in \mathbb {R}^{1 \times D}$ is the feature of the center point $p_{c}$ aggregated from neighboring features. A CBR layer is then applied to $p_{c}$ for positional encoding, yielding $p_{c}^{c} \in \mathbb {R}^{1 \times D}$:

21 $$\begin{aligned} f_{c}^{a}=CBR(f_{c}^{a}). \end{aligned}$$

The same process is employed for the subsequent stages of the feature extractor, continuously downsampling and aggregating local features until the final point cloud representation for classification tasks is obtained.

### Positional difference self-attention

To enhance computational efficiency, a positional difference self-attention mechanism is introduced, which modifies the conventional attention computation by replacing inner-product similarity with element-wise subtraction. Given an input feature set ${\mathscr {F}}_{in}$, the Query, Key, and Value matrices are computed using shared linear transformations:

22 $$\begin{aligned} Q = W_q \mathscr {F}_{in}, \quad K = W_k \mathscr {F}_{in}, \quad V = W_v \mathscr {F}_{in}. \end{aligned}$$

Unlike conventional transformers, the attention weights are computed based on element-wise subtraction rather than dot-product similarity:

23 $$\begin{aligned} \mathscr {A} = {Softmax}(Q - K). \end{aligned}$$

Since element-wise subtraction operates in *O*(*N*) complexity, this formulation significantly reduces computational cost compared with traditional attention mechanisms, which scale as $O(N^2)$. The attention output is then computed as:

24 $$\begin{aligned} {\mathscr {F}}_{sa} = \mathscr {A} \odot V, \end{aligned}$$

where $\odot$ denotes the Hadamard product. To stabilize feature propagation and enhance representation learning, a residual connection is incorporated. The self-attention features ${\mathscr {F}}_{sa}$ are refined through a convolution, batch normalization, ReLU (CBR) module before being merged with the input features:

25 $$\begin{aligned} {\mathscr {F}}_{out} = {\mathscr {F}}_{in} + CBR({\mathscr {F}}_{in} - {\mathscr {F}}_{sa}). \end{aligned}$$

This formulation ensures that local geometric relationships are effectively preserved while improving computational efficiency. The overall complexity of this self-attention mechanism is reduced to *O*(*ND*), making it significantly more scalable for large-scale point cloud processing.

## Experience

The effectiveness of the proposed PointGA method was comprehensively evaluated across multiple tasks and datasets. For point cloud classification, experiments were conducted on the most challenging variant, PB_T50_RS, of the real-world scanned dataset ScanObjectNN^48^, as well as on the standard synthetic dataset ModelNet40^49^. In the semantic segmentation task, the Stanford Large-Scale 3D Indoor Spaces Dataset^50^ (S3DIS), which includes rich scene data, was utilized for evaluation. Furthermore, extensive ablation studies were performed to validate the rationale behind the selected parameters and the designed modules.

### Shape classification

#### Data and metric

In this study, ScanObjectNN and ModelNet40 are employed as datasets to evaluate the classification performance of the proposed model. ScanObjectNN is derived from 3D scans of real-world scenes, incorporating common noise and incompleteness encountered during scanning, thereby closely simulating the model’s effectiveness in real-world applications. The dataset comprises 14,802 real-world scanned objects across 15 categories, exhibiting a significant long-tailed distribution with a Gini coefficient of 0.62. The number of samples per category ranges from 230 (monitors) to 1380 (tables), resulting in a maximum-to-minimum ratio of 6:1. This characteristic makes it particularly suitable for investigating the robustness of point cloud classification models in real-world applications. Furthermore, ScanObjectNN provides multiple versions, and we conducted experiments on its most challenging variant, PB_T50_RS, strictly following the official training/testing split (80%/20%). ModelNet40 is a classical benchmark dataset for 3D object classification, consisting of 12,311 high-precision CAD models across 40 semantic categories. The dataset exhibits an approximately uniform distribution (standard deviation $\sigma = 27.6$), with the largest category (plant) containing 315 samples and the smallest category (flower_pot) containing 171 samples, resulting in a maximum-to-minimum ratio of 1.8:1. The standard split of the dataset was adopted in our experiments, with 9843 samples for training and 2468 samples for testing. The significant inter-class variation and intra-class diversity within this dataset provide an ideal platform for validating feature extraction and classification algorithms for geometric shapes. To ensure fairness in evaluation, Overall Accuracy and Mean Per-Class Accuracy (mAcc) are used as metrics, and a voting mechanism is not employed, as it is impractical for real-world applications.

#### Implementation details

All training and testing procedures in this study were conducted on a workstation equipped with two NVIDIA 2080TI GPUs, an Intel(R) Core(TM) i9-10900K CPU @ 3.70GHz, and 64GB of memory. For the ScanObjectNN dataset, the model was trained for 200 epochs using the AdamW optimizer^51^, with an initial learning rate of 0.002 and a weight decay factor of 0.05. The learning rate was progressively reduced to 0.0001 via the Cosine Annealing Learning Rate Scheduler (CosineLRScheduler). The input data consisted of 1024-point clouds, with a batch size of 32. The loss function used was cross-entropy loss with label smoothing, with a smoothing rate set to 0.5. For the ModelNet40 dataset, the model was trained for 250 epochs using the SGD optimizer^52^, with an initial learning rate of 0.1 and a weight decay factor of 0.0002, while other parameters remained unchanged. Additionally, several common data augmentation techniques were applied, including height augmentation, random translation within the range of [− 1/5, 1/5], and random scaling within the range of [2/3, 3/2].

**Table 1:** Performance comparison on ScanObjectNN datasets. The table presents metrics including overall accuracy (OA, %), mean per-class accuracy (mAcc, %), and parameter count (Param.).

Method	Input	#points	OA(%)	mAcc(%)	Param.
PointNet^20^	xyz	1024	68.2	63.4	3.47M
PointNet++^21^	xyz, nr	5000	77.9	75.4	1.74M
DGCNN^22^	xyz	1024	78.1	73.6	21.00M
RepSurf-U^53^	xyz	1024	84.3	81.3	**1.48M**
PointCMT^54^	xyz	1024	86.7	84.8	12.60M
Point-M2AE^55^	xyz	1024	86.4	–	–
Point-LGMask^56^	xyz	1024	85.3	–	–
DualMLP^57^	xyz	1024	86.4	–	14.30M
Point-BERT^41^	xyz	8192	83.1	–	–
PointMLP^24^	xyz	1024	85.4	83.9	12.60M
PointGL^58^	xyz	1024	86.9	85.2	4.16M
PointStack^59^	xyz	1024	87.2	86.2	–
Ours	xyz	1024	**87.6**	**86.4**	1.73M

Significant values are in bold.

#### Classification on ScanObjectNN dataset

The experimental results on the ScanObjectNN dataset are presented in Table 1. On the most challenging variant, PB_T50_RS, our method achieved an overall accuracy of 87.6% and a mean class accuracy of 86.4%. Compared with other methods, PointGA demonstrated superior performance, indicating that the model effectively handles real-world challenges such as noise and missing data. In terms of overall accuracy, PointGA outperformed the classical PointNet^20^ and PointNet++^21^ by 19.4% and 9.7%, respectively. Unlike PointNet, which employs a simple global feature aggregation mechanism, and PointNet++, which enhances local neighborhood information through hierarchical learning, PointGA further integrates geometric-aware information to improve feature representation. It is worth noting that PointNet++ utilized 5000 points with normal vectors as input, while Point-BERT^41^ used 8192 points for each object. In contrast, PointGA only used 1024 points without normal vectors. Despite the significantly reduced number of points and input features, PointGA still surpassed PointNet++ and Point-BERT in classification accuracy. This demonstrates that PointGA achieves more efficient feature extraction and classification, leveraging its geometric-aware attention mechanism to capture discriminative patterns in the presence of occlusion and noise. For mean class accuracy, PointGA also outperformed state-of-the-art models such as PointGL^58^ and PointStack^59^ by 1.2% and 0.2%, respectively. While PointGL incorporates graph-based learning and PointStack utilizes hierarchical stacking, they still struggle to balance performance across different object categories. PointGA, with its geometric-aware design, achieves more consistent recognition across diverse object classes, mitigating the issue of biased predictions. In terms of parameter efficiency, PointGA contains only 1.73M parameters, representing approximately 91.8% fewer parameters than DGCNN’s^22^ 21.0M and 87.9% fewer than DualMLP’s^57^ 14.30M. Although PointGA has 0.26M more parameters than RepSurf-U^53^, the resultant performance improvement is significant, which is considered a worthwhile trade-off. This highlights PointGA’s ability to achieve competitive performance with fewer computational resources, making it a promising solution for real-world applications.

**Table 2:** Performance comparison on ModelNet40 datasets. The table presents metrics including overall accuracy (OA, %), mean per-class accuracy (mAcc, %), and parameter count (Param.).

Method	Input	#points	OA(%)	mAcc(%)	Param.
PointNet^20^	xyz	1024	89.2	86.0	3.47M
PointNet++^21^	xyz, nr	5000	91.9	–	1.74M
DGCNN^22^	xyz	1024	92.9	90.2	21.00M
PointCNN^60^	xyz, nr	1024	92.5	–	–
PointWeb^61^	xyz, nr	1024	92.3	89.4	–
KPConv^23^	xyz	1024	92.9	–	14.30M
Point Transformer^40^	xyz	1024	93.7	90.6	–
PCT^39^	xyz	1024	93.2	–	2.88M
Point-BERT^41^	xyz	8192	93.8	–	–
PointMLP *^24^	xyz	1024	**94.1**	**91.3**	12.60M
PointGL^58^	xyz	1024	93.0	90.4	4.16M
PointConT^62^	xyz	1024	93.5	–	–
DualMLP^57^	xyz	1024	93.7	–	14.30M
Ours	xyz	1024	93.8	90.9	**1.73M**

Significant values are in bold.

#### Classification on ModelNet40 dataset

To ensure a more accurate and fair assessment of the proposed model, evaluations were also conducted on the standard CAD object dataset, ModelNet40, with the results presented in Table 2. In comparison with various state-of-the-art methods, PointGA achieved remarkable results, recording an overall accuracy of 93.8% and a mean class accuracy of 90.9%. While PointMLP^24^ attained the highest accuracy through the use of a voting mechanism and a substantial parameter count of 12.6M, it is crucial to acknowledge that real-world applications often require careful consideration of computational efficiency and resource constraints. The reliance on a large number of parameters and an ensemble-like voting strategy introduces challenges in terms of deployment and inference speed. In contrast, PointGA achieves competitive accuracy without resorting to such resource-intensive strategies, demonstrating its efficiency in real-world scenarios. By using only 1024 points as input per object, our model achieved an overall accuracy comparable to Point-BERT^41^, which leveraged a transformer-based architecture with 8192 points as input. This highlights the effectiveness of PointGA in efficiently extracting representative features with significantly fewer input data, reducing computational overhead while maintaining strong recognition capabilities. Even when compared with the latest models such as DualMLP^57^, PointConT^62^, and PointGL^58^, PointGA continued to achieve superior overall accuracy. Although DualMLP’s performance is close to ours, it is noteworthy that our model operates with only 1.73M parameters, representing an 87.9% reduction compared to DualMLP’s 14.3M. This highlights that PointGA significantly reduces model complexity and resource demands while maintaining competitive classification performance. Such efficiency makes it a promising choice for scenarios where computational resources are limited but high accuracy remains a priority.

#### Visualization

**Fig. 4:**
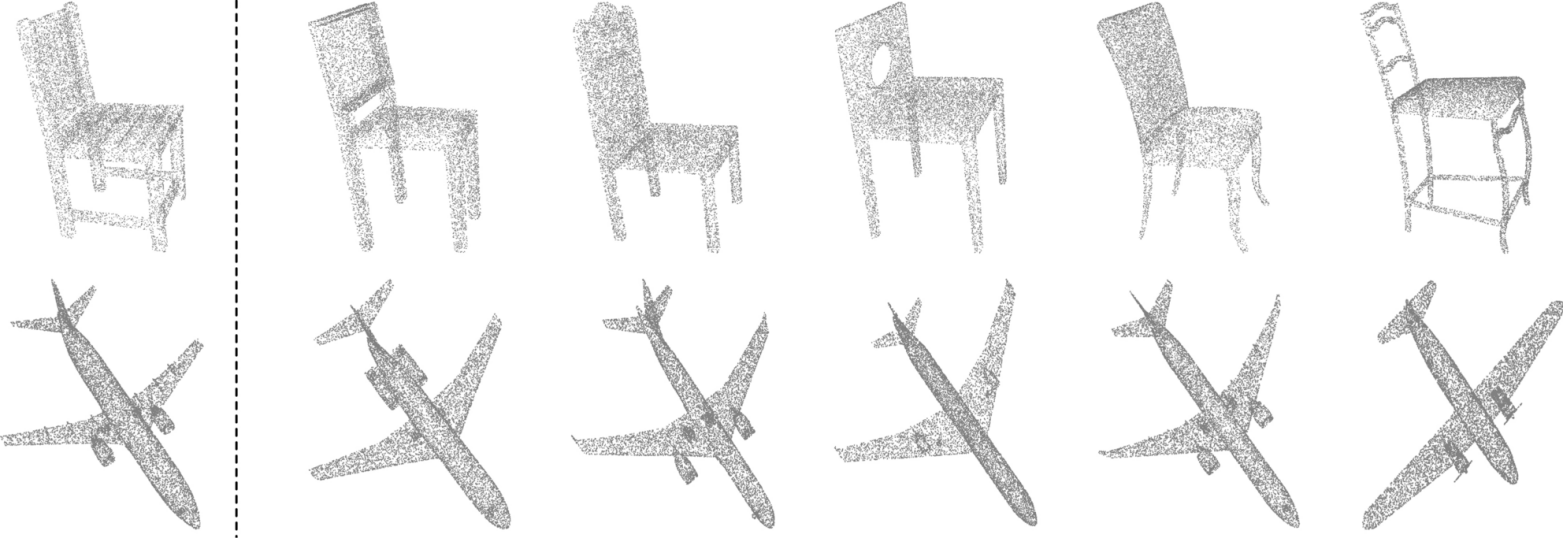
Visualization of shape retrieval on the ModelNet40 dataset. The leftmost column represents the query shape, while the models retrieved based on feature similarity are displayed to the right of the dashed line.

To validate the feature representation capability learned by the PointGA model, an experiment was designed based on feature space retrieval. Specifically, a point cloud object’s feature representation, processed by PointGA, was randomly selected as the query feature. Subsequently, several nearest neighbor features from the entire feature space that exhibited the highest similarity to the query feature were retrieved. In Fig. 4, the left side shows the shape of the query object, while the right side displays multiple objects retrieved through nearest neighbor search in the feature space that are most similar to the query object. From the visualization results, it can be observed that, although some retrieved shapes exhibit differences in details (such as the legs of a chair or the wings of an airplane), the overall shape and semantic features remain highly similar to the query object. This demonstrates that the PointGA model possesses strong feature representation capabilities in preserving geometric structure and semantic information, effectively capturing both local and global geometric information within point cloud data, and accurately clustering similar objects in the feature space. This feature space retrieval analysis further proves the robustness of PointGA across various geometric structures and object categories.

#### Computational efficiency analysis

Table 3 presents the computational cost and inference speed of various methods. Our method demonstrates a favorable balance between accuracy and efficiency, achieving 1.73M parameters, 1.97G FLOPs, and an inference time of 27.4ms. Compared to DGCNN (2.43G FLOPs, 118.6ms) and KPConv (203.2ms), our method significantly reduces inference time while maintaining a lower computational burden. While PointNet exhibits the lowest FLOPs (0.45G) and the fastest inference time (17.1ms), it struggles to deliver competitive accuracy, limiting its applicability in complex scenarios. On the other hand, RepSurf-U achieves a minimal parameter count (1.48M) and relatively low FLOPs (0.9G), but its inference time (155.9ms) is considerably higher, indicating potential inefficiencies in computation or memory access. In contrast, our method strikes a more balanced approach by maintaining low parameter count and computational complexity while ensuring faster inference and higher accuracy. This advantage is primarily attributed to our Position Differential Attention (PDA) mechanism, which leverages linear complexity to model geometric relationships effectively. PDA’s design avoids the quadratic complexity of traditional self-attention mechanisms, enhancing both efficiency and accuracy. These findings highlight our method’s suitability for real-time point cloud processing, offering a pragmatic solution that balances speed, resource efficiency, and performance.

**Table 3:** Computational cost and inference speed of different methods. The table presents metrics including Floating point operations (FLOPs), inference time.

Method	Param.	FLOPs	Inference time
PointNet	3.47M	**0.45G**	**17.1ms**
PointNet++	1.74M	1.68G	78.5ms
DGCNN	21.00M	2.43G	118.6ms
RepSurf-U	**1.48M**	0.9G	155.9ms
PCT	2.88M	2.32G	37.7ms
KPConv	14.30M	-	203.2ms
PointMLP	12.60M	15.67G	60.4ms
PointGL	4.16M	0.63G	32.7ms
Ours	1.73M	1.97G	27.4ms

Significant values are in bold.

**Fig. 5:**
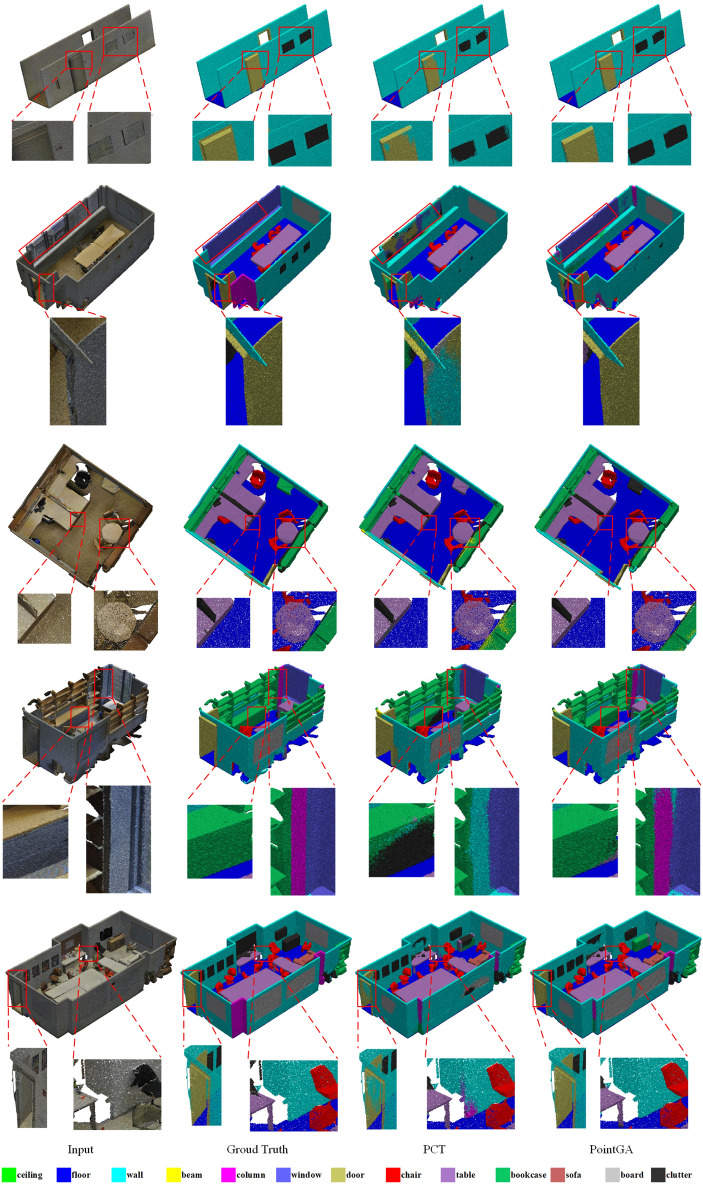
Comparison of semantic segmentation effect of S3DIS. The first column shows the input point cloud in the original color, the second is the PCT result, the third is the Ground Truth, and the fourth is the PointGA result, with a dotted red box marking the difference between the PCT and PointGA, and zooming in to see the details.

### Semantic segmentation

#### Data and metric

The segmentation capability of the PointGA model was evaluated using the S3DIS dataset. This dataset is derived from real-world scans of six different buildings, encompassing 3D point cloud data of over 200 rooms, and includes label information for 13 semantic categories, such as walls, floors, tables, chairs, doors, and bookshelves, among other common indoor objects. The diverse semantic categories and varied environments present both challenges and comprehensiveness for model training and testing, enabling a thorough assessment of PointGA’s semantic segmentation performance in complex indoor settings. To comprehensively compare the performance of different models, we followed the standard evaluation protocol and selected the most challenging scene, Area 5, for testing. Three commonly used evaluation metrics were reported: Overall Accuracy (OA), Mean Per-Class Accuracy (mAcc), and Mean Intersection over Union (mIoU). These metrics provide a holistic evaluation of the strengths and weaknesses of the models in the semantic segmentation task. Additionally, the classification accuracy for each of the 13 semantic categories was detailed.

#### Implementation details

In the implementation on the S3DIS dataset, the number of input points per sample was set to 4096. The optimizer employed was AdamW, with an initial learning rate of 0.001 and a weight decay parameter of 0.05. The loss function used was cross-entropy loss. For a fair comparison, all models were trained for 100 epochs with a batch size of 16. Additionally, the input data consisted of nine dimensions, including the original xyz coordinates, RGB color values, and normalized xyz coordinates. A cosine annealing learning rate scheduler was also employed to dynamically adjust the learning rate, aiming to enhance training efficiency and model convergence speed.

#### Semantic segmentation on S3DIS dataset

PointGA demonstrated outstanding performance in the semantic segmentation task on the S3DIS dataset, outperforming many existing state-of-the-art methods. As shown in Table 4, although PointGA’s overall accuracy (OA) of 88.9% still falls slightly short compared to BAAF-Net, it achieved superior results in mean accuracy (mAcc, 74.4%) and mean Intersection over Union (mIoU, 66.2%). This suggests that PointGA provides a more balanced classification performance across multiple categories.

The exceptional performance of PointGA can be attributed to its ability to incorporate geometric information expansion, trigonometric positional encoding, and positional difference attention. By integrating neighborhood geometric information such as direction vectors and curvature, PointGA enhances the perception of local geometric structures, thereby improving the ability to distinguish between geometrically similar but semantically different objects, such as *table* and *bookshelf*, which contributes to an increased mAcc. Furthermore, the use of trigonometric positional encoding strengthens the positional representation of points in the feature space, allowing for a more precise delineation of complex object boundaries. This enhancement is particularly beneficial for categories such as *door* and *sofa*, where capturing intricate structures leads to improved IoU scores. Additionally, the positional difference attention mechanism refines feature extraction by leveraging relative positional information, enabling a more effective distinction of subtle variations in local structures. This approach proves advantageous in recognizing categories with ambiguous boundaries, such as *clutter*, ultimately contributing to a higher overall mIoU.

Compared to PCT, which employs standard self-attention mechanisms, PointGA leverages geometric-aware feature extraction, enabling better fine-grained feature learning. This is particularly evident in complex indoor objects such as doors, tables, sofas, and bookcases, where PointGA achieved the highest IoU among all comparison methods. Unlike PointNet++, which relies solely on local neighborhood aggregation, PointGA effectively captures both local geometric details and global structural patterns, leading to superior recognition of objects with intricate structures.

Moreover, for categories like *clutter*, which contain highly irregular and diverse shapes, many models struggle due to the blurred boundaries and heterogeneous characteristics of these objects. However, PointGA, with its enhanced feature extraction capability, effectively distinguishes subtle variations, achieving 56.6% IoU, surpassing methods such as PointCNN and KPConv. Most models exhibit poor segmentation accuracy for the *beam* and *column* classes, primarily due to their low representation in the dataset, accounting for only 0.044% and 0.127% of the total points, respectively. This limited data availability restricts the learning process. Additionally, their linear structures are prone to misclassification as ceiling or wall classes, a challenge also faced by methods like PointNet++ and KPConv. While PointGA does not completely eliminate these issues, its ability to capture geometric relationships helps mitigate misclassification to a certain extent.

As illustrated in Fig. 5, a comparison of the segmentation results between PointGA and PCT on the S3DIS dataset is presented, with the differences highlighted using red boxes. The visualized results clearly show that PointGA excels in capturing local geometric details and overall structural patterns, producing segmentation results that are closer to the ground truth and significantly better than PCT. This further validates PointGA’s advantage in semantic segmentation tasks.

**Table 4:** Performance comparison on S3DIS datasets (evaluation in Area 5). The table presents metrics including overall accuracy (OA, %), mean per-class accuracy (mAcc, %), mean intersection over union (mIoU, %).

Method	OA	mAcc	mIoU	ceil.	floor	wall	beam	colu.	wind.	door	table	chair	sofa	book.	board	clut.
	(%)	(%)	(%)													
PointNet^20^	-	49.0	41.1	88.8	97.3	69.8	0.1	3.9	46.3	10.8	59.0	52.6	5.9	40.3	26.4	33.2
PointNet++^21^	83.1	63.0	52.9	90.2	96.8	75.1	0.0	5.8	56.8	17.3	68.4	72.0	44.6	61.5	56.9	42.4
PointCNN^60^	85.9	63.9	57.3	92.3	98.2	79.4	0.0	17.6	22.8	62.1	74.4	80.6	31.7	66.7	62.1	56.7
PointWeb^61^	87.0	66.6	60.3	92.0	**98.5**	79.4	0.0	21.1	59.7	34.8	76.3	**88.3**	46.9	69.3	64.9	52.5
PCT^39^	-	67.7	61.3	92.5	98.4	80.6	0.0	19.4	61.6	48.0	76.6	85.2	46.2	67.7	67.9	52.3
VMVF^63^	-	-	65.4	92.9	96.9	**85.5**	**0.8**	**23.3**	65.1	45.7	85.8	76.9	63.1	74.6	**82.1**	57.0
BAAF-Net^64^	**88.9**	73.1	65.4	92.9	97.9	82.3	0.0	23.1	**65.5**	64.9	87.5	78.5	61.4	70.7	68.7	**57.2**
PointGL^58^	88.6	71.9	65.6	–	–	–	–	–	–	–	–	–	–	–	–	–
Ours	88.3	**74.4**	**66.2**	**93.0**	98.4	80.9	0.0	17.5	54.4	**70.9**	**89.4**	80.8	**73.4**	**75.1**	70.4	56.6

## Ablation study

In this section, the analysis focuses on the impact of various components of PointGA on its performance. By systematically removing or replacing different modules and parameters, individual contributions to the model’s accuracy were assessed. All baseline parameter settings and evaluation metrics were implemented under the same configuration as that used in the ScanObjectNN classification experiments.

### Neighbor number *k*

The appropriate number of neighboring points, $k$, is critical for the model’s performance, especially in real-world applications where computational efficiency and the ability to generalize to diverse scenarios are essential. To investigate this, the initial experiments focused on tuning the number of neighbors, $k$. As shown in Table 5, our experiments reveal that the model achieves optimal prediction performance when $k$ is set to 40. Smaller values of $k$ (e.g., $k=20$ or $k=30$) may result in insufficient local information, limiting the network’s ability to capture fine-grained geometric details. In practical scenarios such as autonomous driving or robotic navigation, where accurate and detailed spatial understanding is required, reducing $k$ might hinder the model’s ability to identify and distinguish key features like obstacles or structural components. On the other hand, larger values of $k$ (e.g., $k=50$ or $k=60$) could introduce irrelevant distant neighbor information and additional noise, which in turn would degrade the model’s performance. In real-time applications, excessive neighborhood size increases computational complexity and memory usage, which could be a limiting factor for embedded systems with limited resources.

**Table 5:** Ablation study: number of local neighborhoods. The table presents metrics including overall accuracy (OA, %), mean per-class accuracy (mAcc, %).

*k*	Input	#points	OA(%)	mAcc(%)
20	xyz	1024	86.2	85.5
30	xyz	1024	87.1	85.9
40	xyz	1024	**87.6**	**86.4**
60	xyz	1024	86.8	85.7

Significant values are in bold.

### Dimensions of geometric information expansion

In this ablation study, different geometric features were progressively introduced to evaluate their impact on the model’s performance. As shown in Table 6, the initial 3D coordinates $P_i$ provided a stable starting point for the model, achieving an OA of 86.9%. The overall performance slightly improved with the introduction of simple directional vector information. When normal vector and curvature information were added, increasing the feature dimension to 13, there was a significant improvement in both OA and mAcc. Further introducing distance information $d_1$ and $d_2$, bringing the dimension to 15, resulted in the highest OA of 87.6% and mAcc of 86.4%. However, when additional geometric information ($Q_1, Q_2$) is introduced, the model’s performance declines. This is because prematurely aggregating neighboring point coordinates weakens the geometric characteristics of individual points. In point cloud processing, the unique geometric attributes of each point are crucial for distinguishing different structures. When $Q_1$ and $Q_2$ are directly aggregated into the features of the central point $P_i$ at an early stage, the feature representations of neighboring points become overly similar, reducing the model’s sensitivity to local geometric details. Consequently, the model struggles to effectively capture and differentiate subtle yet critical geometric variations, ultimately affecting overall performance.

**Table 6:** Ablation study: dimensions of geometric information expansion. The table presents metrics including overall accuracy (OA, %), mean per-class accuracy (mAcc, %).

Geometric information extension	Dimension	OA(%)	mAcc(%)
$P_i$	3	86.9	86.0
$P_i$, ${\vec {V}_1}$, ${\vec {V}_2}$	9	87.2	86.2
$P_i$, ${\vec {V}_1}$, ${\vec {V}_2}$, ${\vec {n}_i}$, ${\kappa _i}$	13	87.5	86.3
$P_i$, ${\vec {V}_1}$, ${\vec {V}_2}$, ${\vec {n}_i}$, ${\kappa _i}$, ${d_1}$, ${d_2}$	15	**87.6**	**86.4**
$P_i$, ${\vec {V}_1}$, ${\vec {V}_2}$, ${\vec {n}_i}$, ${\kappa _i}$, ${d_1}$, ${d_2}$, ${Q_1}$, ${Q_2}$	21	87.2	86.1

Significant values are in bold.

### Attention operator

The impact of various attention mechanisms on the model’s performance was investigated, with the results presented in Table 7. Initially, the attention mechanism was removed from the model, employing only MLP, which resulted in an overall accuracy (OA) of 85.3% and a mean class accuracy (mAcc) of 83.2%. Notably, this baseline model achieved the lowest computational cost, with only 1.31 G FLOPs and the fastest inference time of 12.7 ms. Subsequently, scalar attention and vector attention were introduced separately. The results show that using scalar attention improved OA to 85.8% and mAcc to 84.5%, while vector attention further increased OA to 86.7% and mAcc to 85.8%. However, these improvements came at the cost of increased computational complexity, with FLOPs rising to 1.68 G and 2.30 G, respectively, and inference time increasing to 23.0 ms and 41.8 ms. Furthermore, the PCT self-attention mechanism achieved an OA of 87.2% and mAcc of 86.0%, with FLOPs of 2.26 G and inference time of 32.4 ms. Finally, our proposed self-attention operator achieved the best performance, with an OA of 87.6% and mAcc of 86.4%, while maintaining a balance between accuracy and efficiency, with FLOPs of 1.97 G and inference time of 27.4 ms. These results indicate that our proposed self-attention operator not only offers significant advantages in capturing complex features and enhancing classification accuracy but also demonstrates a more favorable trade-off between performance and computational cost compared to other attention mechanisms.

**Table 7:** Ablation study: attention operator. the table presents metrics including overall accuracy (OA, %), mean per-class accuracy (mAcc, %), floating point operations (FLOPs), inference time.

Attention	Input	#points	OA(%)	mAcc(%)	FLOPs	Inference Time
no(MLP)	xyz	1024	85.3	83.2	**1.31**G	**12.7**ms
Scalar attention	xyz	1024	85.8	84.5	1.68G	23.0ms
Vector attention	xyz	1024	86.7	85.8	2.30G	41.8ms
PCT attention	xyz	1024	87.2	86.0	2.26G	32.4ms
Ours attention	xyz	1024	**87.6**	**86.4**	1.97G	27.4ms

Significant values are in bold.

### Frequency scaling factor

Furthermore, to optimize the hyperparameter $\alpha$ in the trigonometric embedding, we drew inspiration from existing positional encoding studies and adopted a progressive tuning strategy to incrementally adjust $\alpha$ and evaluate its impact on model performance, as shown in Table 8. The experimental results indicate that when $\alpha$ is too small, the denominator in the trigonometric encoding function decreases, resulting in high-frequency oscillations. This causes points that are spatially close to exhibit significantly different feature representations, disrupting local continuity and making it difficult for the model to learn smooth geometric structures. As $\alpha$ increases, the frequency of the trigonometric encoding is adjusted to a more appropriate scale, preserving local features while enhancing global information representation. The experimental results show that when $\alpha =1000$, the classification accuracy reaches its peak, suggesting that this value provides an optimal balance between capturing fine-grained details and maintaining global spatial consistency. However, when $\alpha$ becomes excessively large (e.g., 2000 or 3000), the trigonometric function undergoes excessive stretching, causing the encoded values to change more gradually. Consequently, different spatial positions are mapped to similar embeddings, reducing the discriminability of features. This weakens the model’s ability to distinguish geometric variations, ultimately leading to performance degradation. Specifically, when $\alpha =3000$, OA and mAcc drop to 85.1% and 83.0%, respectively. These results suggest that in spatial encoding, the choice of $\alpha$ must be carefully made to balance local feature discriminability and global positional consistency.

**Table 8:** Ablation study: frequency scaling factor $\alpha$. The table presents metrics including overall accuracy (OA, %), mean per-class accuracy (mAcc, %).

$\alpha$	100	500	1000	2000	3000
OA(%)	82.8	87.3	**87.6**	87.4	85.1
mAcc(%)	78.5	86.2	**86.4**	86.2	83.0

Significant values are in bold.

### Pooling strategy

Subsequently, the influence of various feature aggregation methods on the model’s classification performance was investigated. Comparative experiments were conducted using several common pooling strategies, including max pooling, average pooling, and a hybrid approach combining both methods. The results are presented in Table 9. It can be observed that the model’s performance slightly decreased when using max pooling. This is because max pooling primarily retains the most dominant feature responses while potentially discarding subtle yet informative details. When average pooling was used alone, the OA dropped by 0.7%, indicating that relying solely on averaged features tends to smooth variations and may dilute discriminative information. To mitigate these limitations, a hybrid strategy combining max pooling and average pooling was adopted, leveraging the strengths of both approaches. Max pooling captures the most salient features, preserving critical geometric structures, whereas average pooling retains global contextual information and stabilizes feature representation. This pooling strategy is particularly beneficial in point cloud analysis, where objects from different categories exhibit significant geometric variations. Max pooling enhances the representation of key local geometric variations, which is crucial for distinguishing sharp edges, corners, and distinctive surface patterns. Meanwhile, average pooling ensures that the model remains robust by preventing excessive sensitivity to outliers. Experimental results confirmed that this approach achieved the most favorable performance, with the Overall Accuracy (OA) improving to 87.6% and the Mean Class Accuracy (mAcc) increasing to 86.4%.

**Table 9:** Ablation study: pooling strategy. The table presents metrics including overall accuracy (OA, %), mean per-class accuracy (mAcc, %).

Pooling Strategy	OA(%)	mAcc(%)
Max	87.3	86.2
Average	86.9	86.1
Max + Average	**87.6**	**86.4**

Significant values are in bold.

## Discussion

The performance differences observed across datasets, such as ModelNet40 and S3DIS, can be attributed to several key factors, including dataset properties, task alignment, and data quality. Specifically, ModelNet40, which consists of high-quality CAD models with clear geometric structures and no noise, is well-suited for 3D shape learning and classification tasks. On the other hand, S3DIS, composed of real-world indoor point clouds, presents challenges due to noise, occlusions, and varying densities, which make it harder for models to discern clear features for classification. Additionally, the 40 object categories in ModelNet40 exhibit substantial geometric differences, which enhances the performance in classification tasks. In contrast, S3DIS, designed primarily for semantic segmentation, contains categories with overlapping shapes (e.g., tables and chairs), making geometric differentiation more difficult. Lastly, the uniformity and standardization of ModelNet40’s point clouds support more stable feature learning, whereas the varying point cloud density and environmental noise in S3DIS can negatively affect model generalization, thus leading to performance variations.

The proposed PointGA model has demonstrated highly competitive performance on widely used benchmark datasets, showcasing its effectiveness in structured and controlled environments. However, real-world point cloud data often exhibit significant variations in density, noise, and structural complexity, which may pose challenges to the model’s generalization and scalability. To enhance robustness in such scenarios, our future work will explore more adaptive feature aggregation techniques. For instance, granularity-based feature computation has been shown to effectively capture both local and global spatial features in complex environments, potentially improving PointGA’s ability to handle unstructured and unknown point distributions^65^. Another key aspect is the refinement of boundary representation, which is crucial for applications such as autonomous driving and robotic perception. Accurate object boundaries are essential for a precise understanding of the scene, and boundary-aware feature propagation methods have made significant strides in optimizing segmentation results by explicitly modeling geometric discontinuities^66^. Integrating such techniques into PointGA could further enhance its ability to distinguish fine-grained structures in cluttered environments.

## Conclusions

This research introduces PointGA, a Transformer-based model designed to efficiently capture geometric and structural features in point cloud data at a low computational cost, providing robust support for downstream point cloud analysis tasks. Specifically, the geometric information expansion module enhances the representation capability of local neighborhoods and extends the feature set, significantly improving accuracy in classification tasks. In addition, the use of trigonometric positional encoding enriches the model’s positional information encoding capacity, enabling PointGA to exhibit strong robustness across various tasks. Furthermore, the positional difference attention mechanism effectively leverages subtle positional variations within the point cloud, enhancing the perception of complex geometric structures and optimizing performance in classification and segmentation tasks. Experimental results demonstrate the outstanding performance of PointGA on the ScanObjectNN, ModelNet40, and S3DIS datasets. Although the current framework balances accuracy and efficiency, further optimization is necessary to enhance its robustness in real-world scenarios. Future work will explore more adaptive feature aggregation techniques to better handle variations in point cloud density and structural complexity. Additionally, refining boundary-aware feature propagation could improve the model’s ability to capture fine-grained geometric details, which is particularly beneficial for applications requiring precise segmentation. Overall, PointGA establishes a solid foundation for point cloud analysis and shows promising potential in large-scale scene understanding and real-time 3D perception.

## Data Availability

The datasets used in this study are all publicly available. ModelNet40: A widely used benchmark for 3D object classification, consisting of 40 object categories. It can be accessed at https://modelnet.cs.princeton.edu/. ScanObjectNN: A real-world 3D object classification dataset with more challenging scenarios, including occlusions and background noise. The dataset is available at https://hkust-vgd.github.io/scanobjectnn/. S3DIS: A large-scale dataset for 3D semantic segmentation collected from real indoor environments. It is publicly accessible at http://buildingparser.stanford.edu/dataset.html. All figures presented in this paper were created using Microsoft Visio Plan 2 MSO (Version 2501 Build 16.0.18429.20132) 64-bit https://www.microsoft.com/zh-cn/download/office and PyCharm 2024.1.4 (Professional Edition) Build #PY-241.18034.82 https://www.jetbrains.com/pycharm/download/.
